# Cross-cultural comparison of the influence of skin-color change on facial impressions

**DOI:** 10.1177/20416695241288032

**Published:** 2024-10-24

**Authors:** Yuanyuan He, Hiromi Sato, Yoko Mizokami

**Affiliations:** Graduate School of Science and Engineering, 12737 Chiba University, Chiba, Japan; Faculty of Informatics, Graduate School of Informatics, Chiba University, Chiba, Japan; Faculty of Informatics, Graduate School of Informatics, Chiba University, Chiba, Japan

**Keywords:** skin color, facial impression, cultural differences, face perception

## Abstract

Skin color is one of the colors we are most frequently exposed to. It contains information, such as ethnic group and health status, and numerous studies have demonstrated the influence of various facial attributes on the formation of impressions. However, no research has specifically explored the repercussions of treating changes in skin color as a singular variable. We cross-culturally examined skin color changes along with the red-yellow axis and how they influence facial impressions across six face shapes from three types of ethnicities. A 7-point scale was used for evaluation, and the observers evaluated the impression of face images according to the following six evaluation items: healthiness, preference, brightness, whiteness, transparency, and skin tone. The observers were divided into the following four groups: Japan, China, Thailand, and the Caucasus. Differences in the evaluation and association of skin color with various traits emerged between cultures. For instance, East Asian cultures associated positive attributes with reddish skin colors, whereas Caucasians often linked positive traits with yellowish skin colors. These cultural disparities emphasize the dynamic interplay between culture and perception in assessing facial impressions.

In society, interactions with others’ faces are constant and ubiquitous. Considerable research has highlighted the distinctive nature of the human perception of faces compared with other objects. For instance, individuals can swiftly assess facial beauty within remarkably short time frames (Willis & Todorov, 2006). Thorstenson (2018) found that individuals subjectively perceived color differences to be greater on faces than on nonfaces and that the discrimination sensitivity of human facial skin stimulates images in the red direction, which is more potent than that of uniform color images (Hamada et al., 2017). Human faces manifest distinct characteristics attributable to ethnicity, genetics, and environmental upbringing, including skin-color disparity and facial features.

Human skin color varies from black to almost colorless. It is determined by the amount of melanin in the skin, which is in turn determined by genetic and acquired factors (Jablonski & Chaplin, 2000). Moreover, blood circulation causes physiological changes in the skin, and flushing of the surrounding area through the rapid circulation of oxygenated blood (i.e., vasodilatation) usually causes the skin to appear redder and yellower (Changizi & Shimojo, 2011). Sustained physical activity improves skin vascularization, causing the skin to appear redder (Stephen et al., 2009). In contrast, skin vascularization can be impaired by diabetes, high blood pressure, heart disease, and acute illnesses, making the skin appear less red (Changizi & Shimojo, 2011) and making the skin look more yellowish (Coetzee et al., 2009).

Human skin color can convey a plethora of information, including insights into health status, expression, and ethnic background. Skin color and texture are pivotal determinants shaping the impressions of facial appearance. Faces with higher-than-average skin color or texture are perceived as more attractive (Rhodes & Tremewan, 1996; Little et al., 2011; Jones et al., 2007).

Furthermore, the factors affecting facial impressions do not seem to be universal. One study suggested that the effect of skin color on attractiveness may be culturally specific (Han et al., 2018). Chinese participants preferred faces with decreased yellowness, whereas white participants from the UK preferred faces with increased yellowness, possibly because of the differences in economic development and social habits between the two groups. When judging faces of their own race, Chinese observers prefer lighter skin and reduced yellowness, compared with Caucasian observers (Tan & Stephen, 2019). Observers of European descent associated increased yellowness (b*) in Chinese face images with increased attractiveness and health (Lu et al., 2021).

However, prior research has not exclusively isolated the variable of change in skin-color hue. They used the original skin color of the participant in image photography (Lu et al., 2021) or manipulated both the lightness and hue changes (Tan & Stephen, 2019).

Research on skin-color distribution in Japanese women has revealed that yellowish skin has higher colorimetric lightness than does reddish skin (Yoshikawa et al., 2012). In contrast, for Japanese observers, reddish skin appeared brighter than yellowish skin did when both had the same colorimetric lightness. Further, He et al. (2021) showed that this influence of skin-color hue changes in the red-yellow spectrum on the brightness perception of the facial skin tends to be inconsistent depending on the ethnic group to which the observer belongs. However, its impact on other facial impressions has not been thoroughly investigated.

In this study, we used three representative facial profiles and skin colors, focusing solely on variations in the red-yellow spectrum of skin color. The objective of this study was to investigate the impact of skin-color modulation along with the red-yellow axis on facial impressions through tests involving Japanese, Thai, Chinese, and Caucasian individuals. Additionally, we aimed to discern the commonalities and disparities among the observer groups.

## Methods

We conducted an impression evaluation experiment for various face images to investigate how impressions of face stimuli were influenced by skin color modulated along with the red-yellow direction in the CIELAB color space or CIE 1976 L*a*b* color space. The International Commission on Illumination recommended the CIELAB color space in 1976 (CIE 11664–4:2019(E), 2019). It expresses color as three values: L* for perceptual lightness and a* and b* coordinates for hue. a* and b* axes represent the red-green and yellow-blue components, respectively. CIELAB is a nearly uniform color space; therefore, simple Euclidean distances in the spaces can describe the magnitude of the color differences. We can also calculate the hue *h*_ab_ and chroma C*. Conversion of a* and b* to C* and *h*_ab_ is performed as follows:

$$C*=\sqrt{a^{*2}+b^{*2}},\;h_{ab}=a\tan(b*/a*)$$

The observers were divided into the following four groups for international comparisons: Japan, China, Thailand, and the Caucasus.

### Evaluation Items

The evaluation parameters included healthiness, preference, brightness, whiteness, transparency, and skin tone (reddish or yellowish). These specific variables were selected for the following reasons:

1. *Healthiness:* The redness or yellowness of skin complexion often correlates with the body's oxygenation and nutritional state (Changizi & Shimojo, 2011); therefore, this measure can reveal how different skin colors affect perceptions of health.
2. *Preference:* Facial preference is important for communication, and this evaluation should help understand the influence of specific skin colors.
3. *Brightness and whiteness:* Brightness and whiteness were investigated in previous studies (Yoshikawa et al., 2012; He et al., 2021, 2024). The relationships between the metric lightness and perceived whiteness or brightness of facial skin, and between perceived whiteness or brightness of facial skin and other impressions were the main interests of this study. They are among the most significant elements of skin beauty, especially in Asian women.
4. *Transparency:* Evaluating transparency can reflect the lightness, color, clarity, and evenness of the skin, which might influence perceptions of health.
5. *Skin tone:* This item directly relates to the purpose of the experiment, exploring how changes in the red-yellow skin tone continuum affect facial impressions across cultures.

By integrating these diverse evaluation metrics, we analyzed and interpreted variations in the impact of skin color on perceptions of faces across different cultures.

### Stimuli: Generating the Average Face Images

**Figure 1.**
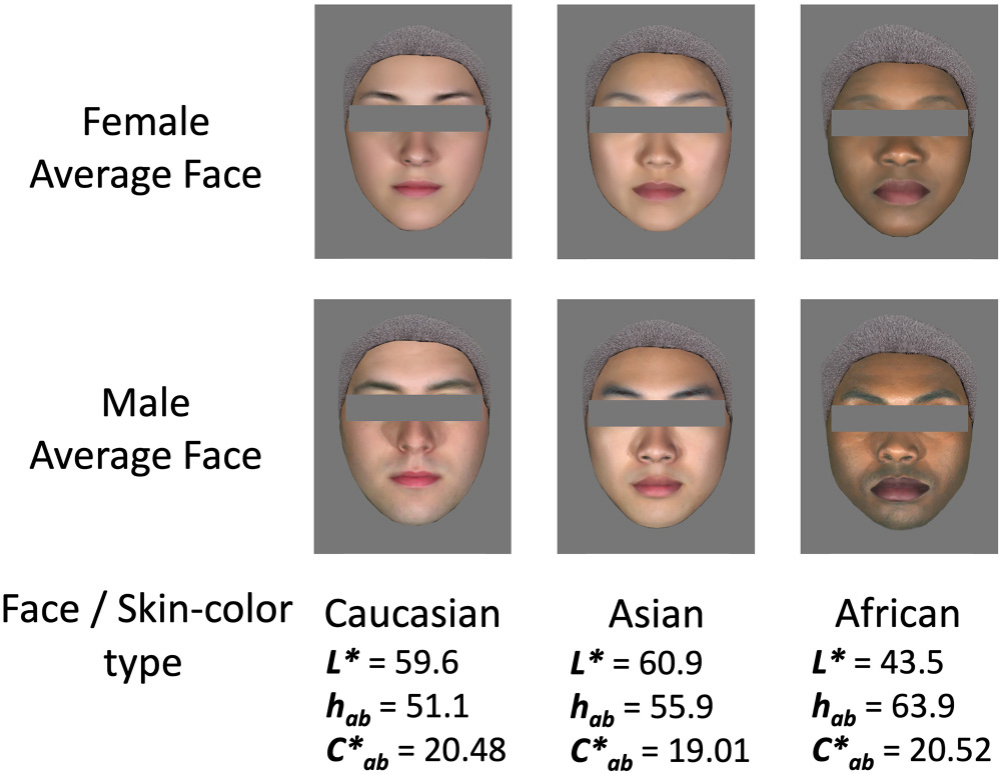
The Caucasian, Asian, and African faces generated with Caucasian, Asian, and African-type skin color, respectively (top: average female face, bottom: average male face). The average CIELAB values (L*, *hab*, and C*) for the three skin-color types were displayed at the bottom.

Three types of facial structures were used in the experiments: Caucasian, Asian, and African. Real facial pictures were sourced from a facial expression database (Yin et al., 2006). We selected 17 female and 16 male faces for Caucasians, 6 female and 10 male faces for Asians, and 4 female and 2 male faces for Africans from the face database to create the average faces. Faces were synthesized using face morphing software (Avrosoft FantaMorph 5). Average male and female faces were created for each skin-color type. The features of each face image were extracted by calibrating the locations of the edges, eyes, corners of the mouth, nose, etc., to generate an average face that could not be assigned to a particular person. A morphed face was used instead of a real face to protect personal information and to eliminate the influence of individual facial features on the evaluation. Noise, such as freckles and spots on the face, was removed, and a hairband at the hairline and an N5 gray bar (Approximate L*, a*, and b* values were 51.6, 0, and 0, respectively) were used to eliminate the effects of factors other than facial color, such as hairstyle, eye color, and expressions. Finally, we generated six average faces for Caucasians, Asians, and Africans, with one female and one male face for each skin-color type. Figure 1 shows the face images generated with the three skin-color types used in the experiment.

### Skin-Color Modification

Six image stimuli were generated by adjusting the color of the skin areas to the average skin color of the three ethnicities, as shown in Figure 1. These values were obtained from previous studies that measured real skin color (Everett et al., 2012; Yoshikawa et al., 2012; Xiao et al., 2017). Color adjustment of the image stimuli was conducted in the same manner as in previous studies (He et al., 2021; Yoshikawa et al., 2012). We modified skin color by changing the L*, a*, and b* ratios and shifted the entire distribution of the facial area along with the a* and b* dimensions. In other words, the color of each pixel in the face area was shifted by the same distance as the shift in the average color while maintaining the color distribution of the face. Only the skin color changed, whereas the eyebrow color, lip color, and luminance distribution were retained in their original form when generating the stimuli.

**Figure 2.**
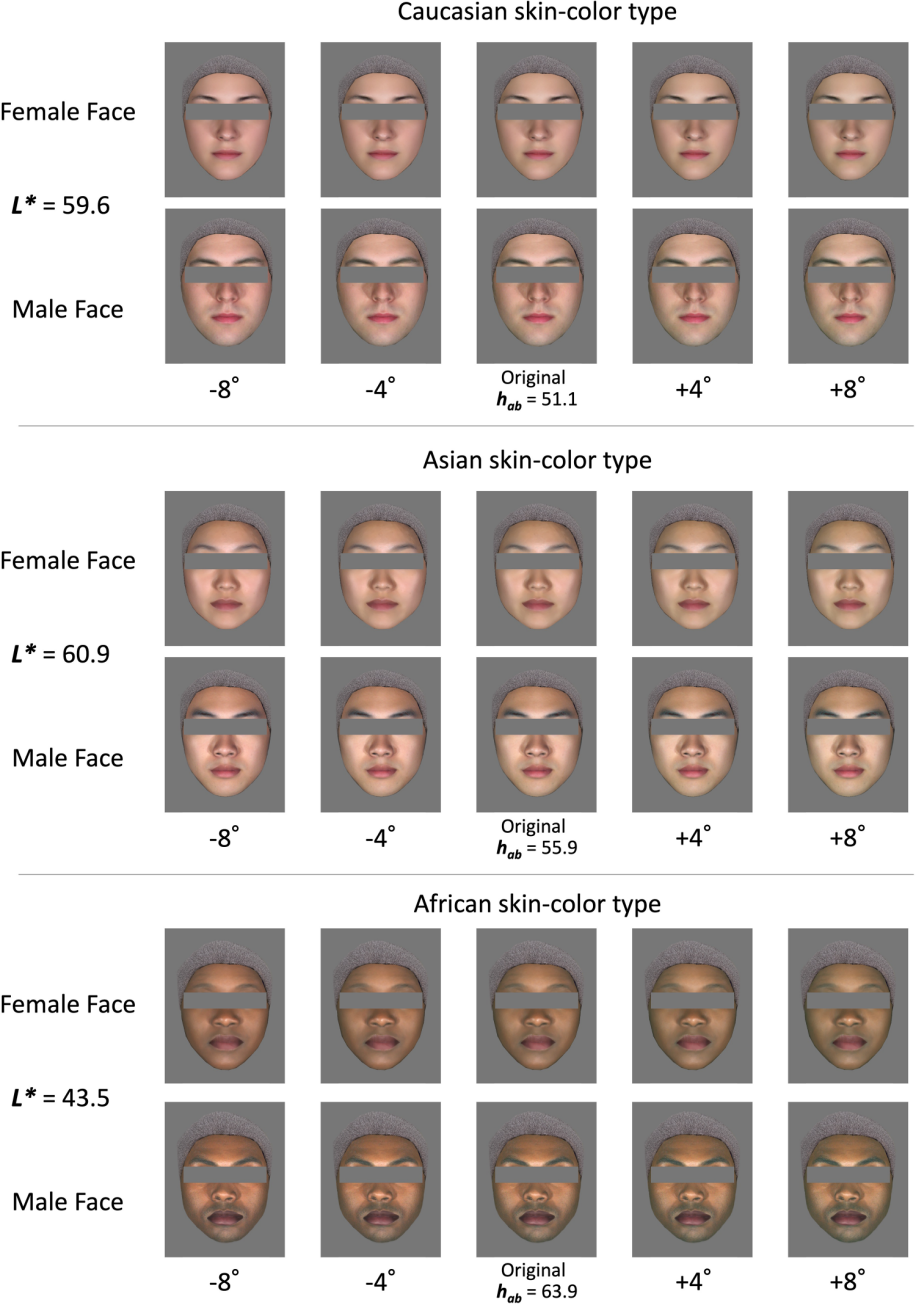
Example of image stimuli. Top: Caucasian skin-color type, middle: Asian skin-color type, bottom: African skin-color type. Each type has the female faces on the top and the male faces on the bottom.

Figure 2 shows examples of stimuli with three skin-color types. Six original face images with constant lightness and different hue levels were used to generate the stimuli. There were five hue levels in total, and the scale of the hue angle *h*_ab_ was in 4-degree steps. The skin color in the middle original image in Figure 2 corresponds to the average CIELAB value for each skin-color type.

### Experimental Environment

Experiments were conducted in Japan, Thailand, the United Kingdom (UK), and France. We used Liquid Crystal displays and a tablet: EIZO ColorEdge CX271, Japan, dark room condition; EIZO ColorEdge CG223 W, Thailand, dark room condition; Apple iPad Pro A1701, UK, Indoor lighting condition, Correlated color temperature of approximately 4500 K; Samsung S24C450 M, France, Indoor lighting condition, Correlated color temperature of approximately 4500 K). All displays were set to a D65 white point and gamma of 1.8. The display was calibrated using a spectroradiometer. The details of the experimental environment are listed in Table 1. Although the display types differed, the visual angle of the stimulus image was maintained at approximately 19° × 13°.

**Table 1.** Details of the Experimental Environment.

Experimental location	Display model	Display resolution	White Luminance	Room color Temperature	Visual angle of stimulus
Japan	EIZO ColorEdge CX271	1920 × 1080 pixels	115 cd/m^2^	–	18.3 × 12.7 °
Thai	EIZO ColorEdge CG223W	1920 × 1080 pixels	114 cd/m^2^	–	18.9 × 13.1 °
United Kingdom	Apple iPad Pro A1701	2224 × 1668 pixels	581 cd/m^2^	4500 K	18.5 × 12.8 °
France	Samsung S24C450M	1920 × 1080 pixels	146 cd/m^2^	4500 K	18.9 × 13.1 °

### Observers

There were 24 Japanese, 12 Chinese, 32 Thai, and 14 Caucasian participants. Observer information is presented in Table 2. The observers in the Caucasian group were British and French. We determined the sample size based on the previous literature on psychophysics (Lu et al., 2021). We also tried calculations to determine the sample size. The effect size was estimated to be medium (Cohen's *d *= 0.5). We aimed to achieve a statistical power of at least 0.80 with a significance level of 0.05. Considering these parameters and using the G*Power software, we calculated that a sample size of *n *= 48 observers would be required to detect the anticipated difference. The experiment was conducted in accordance with the principles of the Declaration of Helsinki (Code of Ethics of the World Medical Association) and the research ethics standards at Chiba University. Informed consent was obtained from all the observers.

**Table 2.** Information of the Observers.

Observer group	Number of observers (Number of females)	Mean age	Test location
Japanese	24 (2)	22.6 (SD = 1.1)	Japan
Chinese	12 (7)	28.4 (SD = 5.3)	UK and Japan
Thai	32 (19)	25.9 (SD = 8.4)	Thai
Caucasian	14 (8)	32.1 (SD = 12.1)	UK and France

### Procedure

**Figure 3.**
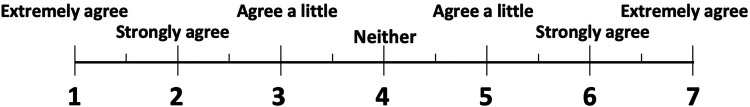
Description of the score.

After confirming that the color vision was normal, using the Ishihara test chart, and recording the observer's basic information, one of the image stimuli with a white frame was randomly displayed at the center of the display with an N5 background. A 7-point scale was used for the evaluation. The observers evaluated the impression of the whole face on a scale of 1–7 based on six evaluation items: healthiness (a score of 1 means that the face is perceived as more unhealthy and a score of 7 means that the face is perceived as more healthy), preference (1-dislike, 7-like), brightness (1-darker, 7-brighter), whiteness (1-lower whiteness, 7-higher whiteness), transparency (1-lower transparency, 7-higher transparency), and skin tone (1-more reddish, 7-more yellowish). Figure 3 shows a description of the scores. The observers assigned scores in increments of 0.5. In total, 30 face image stimuli were presented in randomized order, and each stimulus was evaluated once by all observers.

The instructions for the experiment and evaluation item forms were all in English. For non-native English-speaking observers, native speakers from each country who understood the experimental process provided secondary explanations to observers in their native languages.

The observers were allowed to use interpolation scores, and there was no time limitation for evaluation or stimulus presentation. In the actual experiment, there were no instances in which the observers used a large amount of time (e.g., greater than 1 min for a single scoring session) to think about the responses.

## Results

### Data Analysis

**Figure 4.**
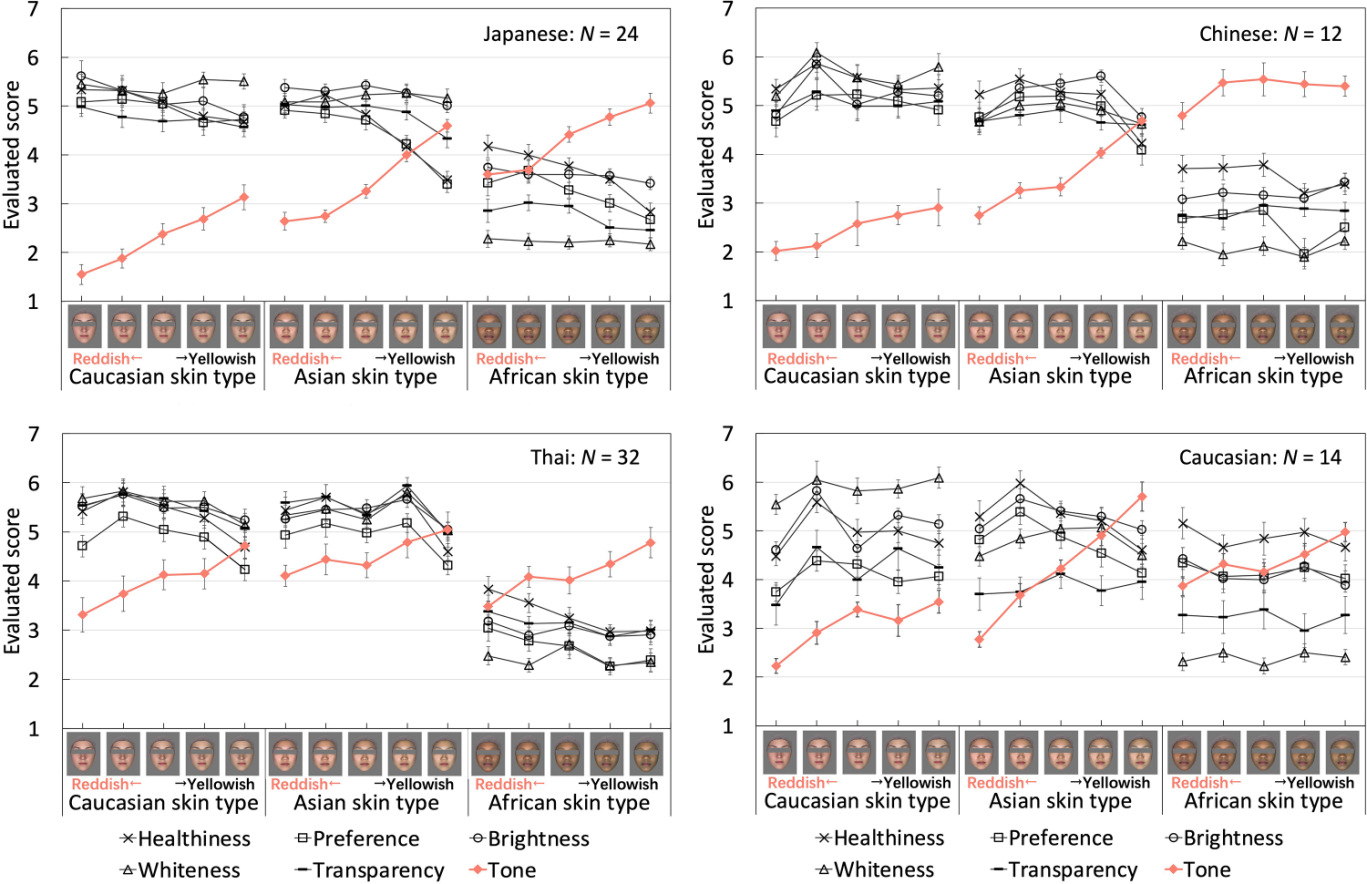
Mean results with standard errors of each group's evaluation scores for the six items for the face stimuli with the three skin-color types. The horizontal axis represents the change in skin color of the image stimuli at five levels (from left to right, the skin color changed from reddish to yellowish) for the three skin-color types. The vertical axis represents the average evaluation results of each group of observers. The red line represents the skin tone evaluation results. Error bars indicate standard errors.

We obtained data from the four observer groups through an impression evaluation experiment in which observers rated six items on a 7-point scale for evaluating 30 faces with three skin-color types. First, we calculated the mean results and standard errors of each group's evaluation scores for the three skin-color types and presented them as line graphs (Figure 4).

**Figure 5.**
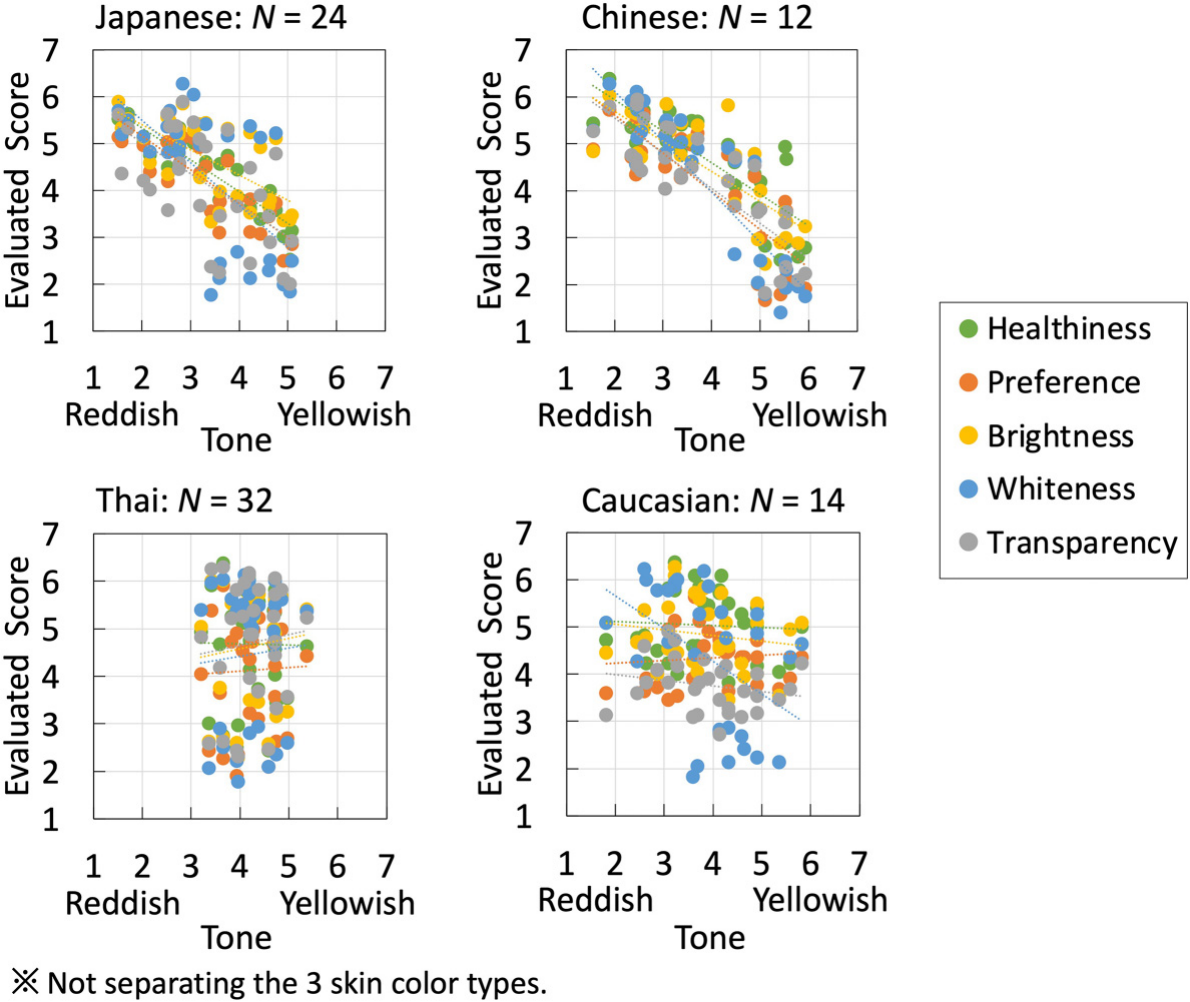
Results of the four observer groups without distinguishing between skin-color types, with the score for skin tone on the horizontal axis and those for the other five items on the vertical axis. Dotted lines represent approximate straight lines.

To compare the overall effects of changes in skin color on impressions without distinguishing between skin-color types, we demonstrated the relationship between skin tone and other item evaluation scores for data using a scatterplot and added linear approximation lines. Figure 5 shows the results of observers’ average scores on the 30 stimulus images presented separately for the four observer groups. In Table 3, we also demonstrate the pairwise relationship among all items. The slope values of the approximate straight lines representing their relationships and correlation coefficients, *r*, are provided for the comparison.

**Table 3.** The Slope of the Relationship Between the Pairwise Average Scores for All Items.^a^

Japanese *N *= 24					
	Healthiness	Preference	Brightness	Whiteness	Transparency
Tone	**−0.7 (*r *= −0.96)**	**−0.74 (*r *= −0.93)**	**−0.46 (*r *= −0.72)**	**−0.94 (*r *= −0.67)**	**−0.70 (*r *= −0.73)**
Healthiness		1.06 (*r *= 0.97)	0.83 (*r *= 0.78)	1.40 (*r *= 0.72)	1.06 (*r *= 0.81)
Preference			0.84 (*r *= 0.87)	1.49 (*r *= 0.85)	1.08 (*r *= 0.90)
Brightness				1.75 (*r *= 0.96)	1.22 (*r *= 0.98)
Whiteness					0.66 (*r *= 0.97)

Chinese *N *= 12					
	Healthiness	Preference	Brightness	Whiteness	Transparency
Tone	**−0.66 (*r *= −0.93)**	**−0.82 (***r** *= −0.88)**	**−0.52 (*r *= −0.82)**	**−1.11 (*r *= −0.91)**	**−0.70 (*r *= −0.90)**
Healthiness		1.29 (*r *= 0.98)	1.04 (*r *= 0.93)	1.64 (*r *= 0.95)	1.05 (*r *= 0.94)
Preference			0.82 (*r *= 0.96)	1.27 (*r *= 0.96)	0.82 (*r *= 0.96)
Brightness				1.48 (*r *= 0.96)	0.96 (*r *= 0.96)
Whiteness					0.64 (*r *= 0.99)

Thai *N *= 32					
	Healthiness	Preference	Brightness	Whiteness	Transparency
Tone	**−0.20 (*r *= −0.09)**	**−0.04 (*r *= −0.02)**	0.11 (*r *= 0.05)	0.14 (*r *= 0.05)	0.07 (*r *= 0.03)
Healthiness		1.06 (*r *= 0.99)	1.09 (*r *= 0.97)	1.36 (*r *= 0.96)	1.10 (*r *= 0.98)
Preference			1.04 (*r *= 0.98)	1.29 (*r *= 0.98)	1.04 (*r *= 0.99)
Brightness				1.24 (*r *= 1.00)	0.98 (*r *= 0.99)
Whiteness					0.79 (*r *= 0.99)

Caucasian *N *= 14					
	Healthiness	Preference	Brightness	Whiteness	Transparency
Tone	**−0.09 (*r *= −0.21)**	0.00 (*r *= 0.00)	**−0.05 (*r *= −0.28)**	**−0.79 (*r *= −0.49)**	**−0.28 (*r *= −0.32)**
Healthiness		0.92 (*r *= 0.88)	1.04 (*r *= 0.68)	0.91 (*r *= 0.24)	0.40 (*r *= 0.32)
Preference			0.80 (*r *= 0.55)	0.46 (*r *= 0.13)	0.13 (*r *= 0.10)
Brightness				1.94 (*r *= 0.80)	0.66 (*r *= 0.80)
Whiteness					0.28 (*r *= 0.84)

a Negative values in the table are marked in bold.

**Figure 6.**
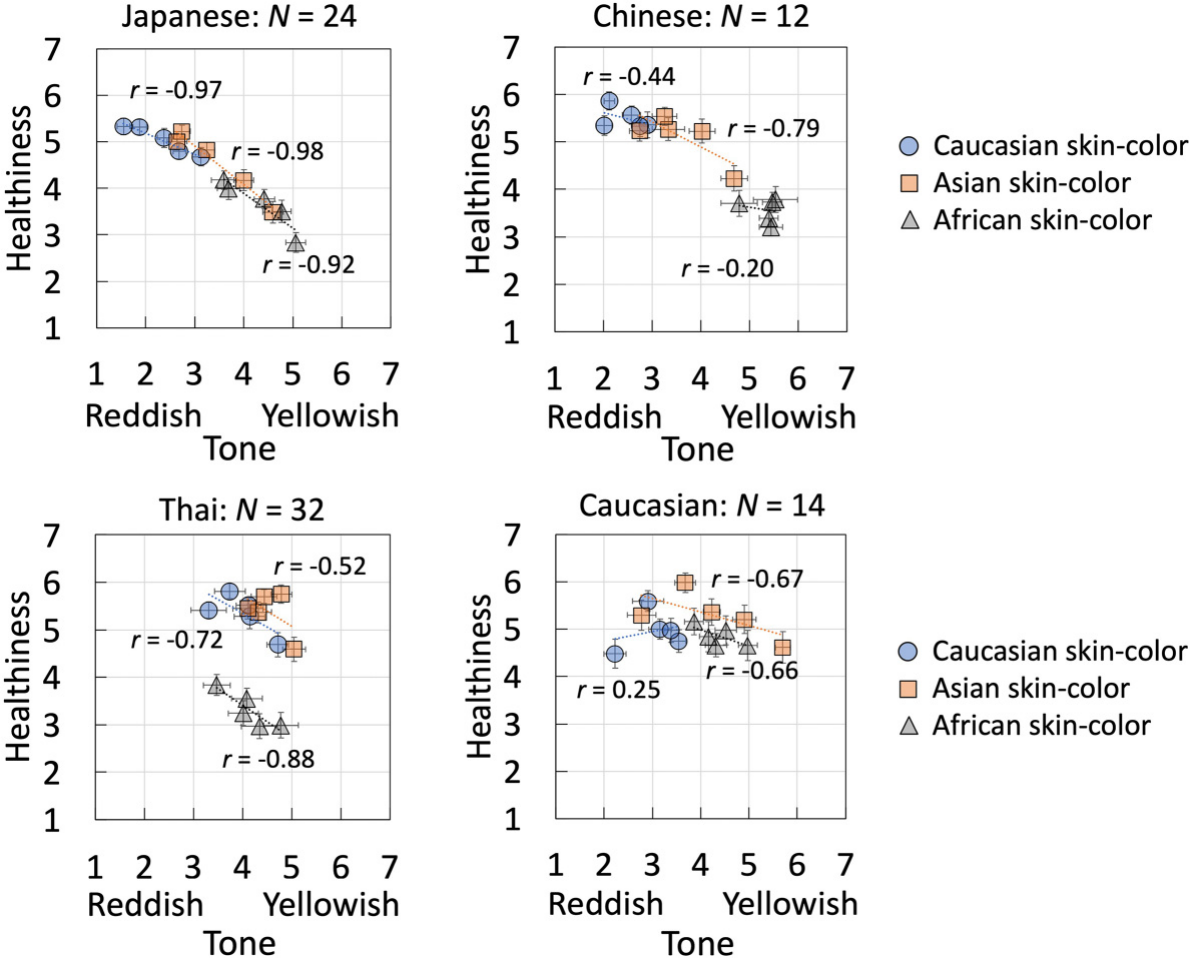
Correlation between the skin tone (reddish-yellowish) and healthiness scores of four groups of observers. Results are shown for each of the three skin-color types. The horizontal and vertical axes represent skin tone and the healthiness scores, respectively. Error bars indicate standard errors.

**Figure 7.**
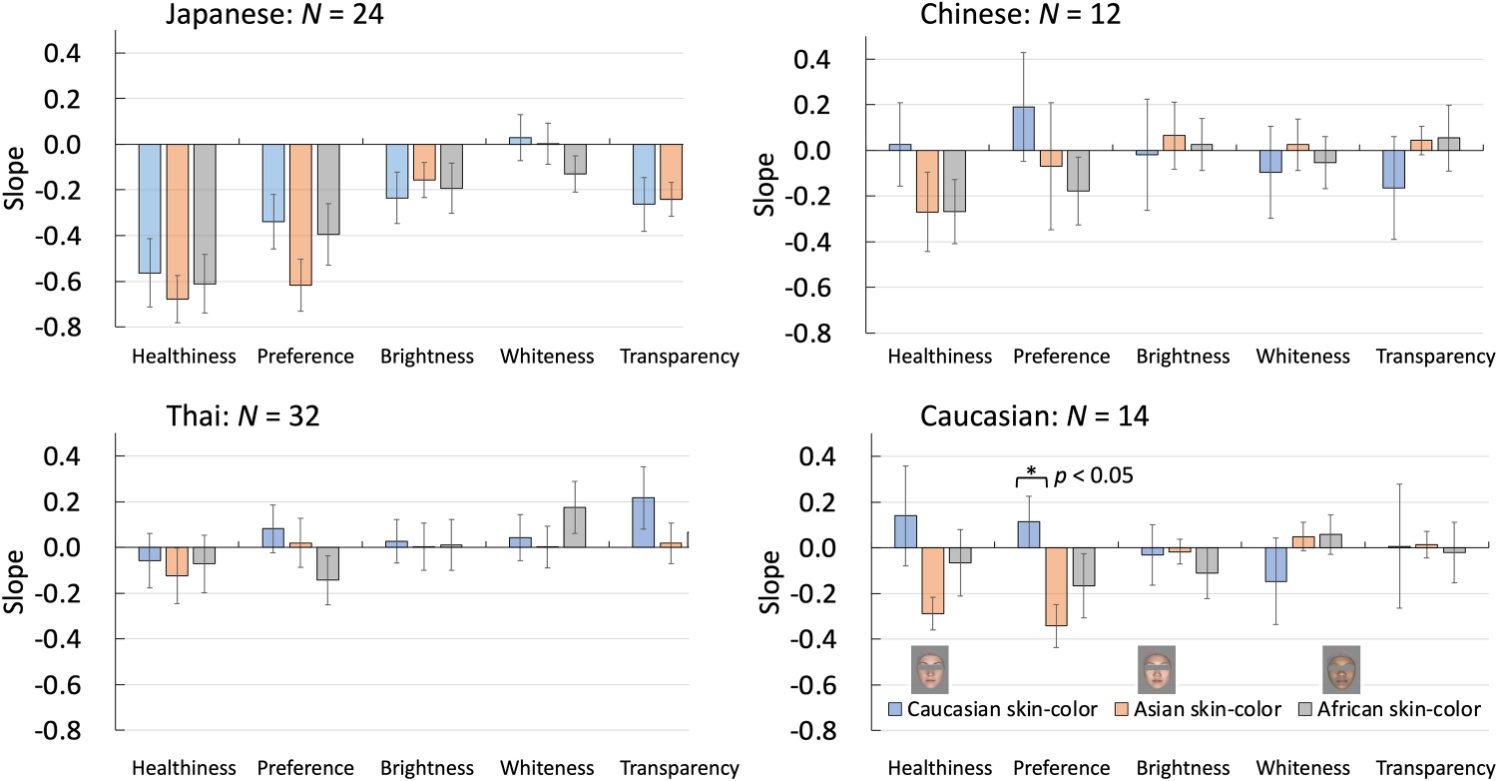
The slope value for the relationship between skin tone and other items for the four observer groups. The three colored bars indicate the three skin-color types. The horizontal axis represents healthiness, preference, brightness, whiteness and transparency. The vertical axis is the slope value calculated for the relationship between the skin tone and other items. Error bars indicate standard errors.

Next, we performed the above for the results of each of the three skin-color types, comparing all items two by two and deriving the slopes of the approximate straight lines of their relationships. Figure 6 shows the relationship between skin tone and healthiness scores, as an example. The correlation coefficients, *r*, are presented in a table (Table 4). The slope values of skin tone with other items are presented as a bar graph (Figure 7). Those slope values of the correlations between skin tone and other item evaluation scores for all observers were used in the analysis of variance (ANOVA).

**Table 4.** The Coefficient of Correlation among All Evaluation Items.^a^

Japanese *N *= 24						
Evaluation item	Stimulus type	Tone	Healthiness	Preference	Brightness	Whiteness
Healthiness	Caucasian	**−0**.**97**				
	Asian	**−0**.**98**				
	African	**−0**.**92**				
Preference	Caucasian	**−0**.**84**	**0**.**94**			
	Asian	**0**.**93**	**0**.**96**			
	African	**−0**.**93**	**0**.**93**			
Brightness	Caucasian	**−0**.**96**	**0**.**86**	**0**.**66**		
	Asian	**−0**.**82**	**0**.**85**	**0**.**93**		
	African	**−0**.**84**	**0**.**94**	**0**.**55**		
Whiteness	Caucasian	0.18	**−0**.**61**	**−0**.**79**	−0.15	
	Asian	**0**.**59**	−0.47	−0.36	0.00	
	African	**−0**.**68**	**0**.**80**	**0**.**59**	**0**.**90**	
Transparency	Caucasian	**−0**.**91**	**0**.**78**	**0**.**57**	**0**.**98**	−0.05
	Asian	**−0**.**86**	**0**.**90**	**0**.**96**	**0**.**94**	−0.12
	African	**−0**.**82**	**0**.**82**	**0**.**92**	**0**.**62**	0.32

Chinese *N *= 12						
Evaluation item	Stimulus type	Tone	Healthiness	Preference	Brightness	Whiteness
Healthiness	Caucasian	−0.44				
	Asian	**−0**.**79**				
	African	−0.20				
Preference	Caucasian	0.23	**0**.**66**			
	Asian	0.42	**0**.**90**			
	African	−0.11	**0**.**95**			
Brightness	Caucasian	−0.08	**0**.**77**	**0**.**63**		
	Asian	0.02	**0**.**56**	**0**.**74**		
	African	0.36	−0.21	0.01		
Whiteness	Caucasian	0.01	**0**.**77**	**0**.**62**	**0**.**87**	
	Asian	−0.29	**0**.**71**	**0**.**89**	**0**.**78**	
	African	**−0**.**51**	0.31	0.47	0.37	
Transparency	Caucasian	−0.06	**0**.**81**	**0**.**57**	**0**.**95**	**0**.**97**
	Asian	**−0**.**51**	**0**.**59**	**0**.**75**	0.24	**0**.**83**
	African	0.46	−0.30	−0.27	0.01	0.05

Thai *N *= 32						
Evaluation item	Stimulus type	Tone	Healthiness	Preference	Brightness	Whiteness
Healthiness	Caucasian	**−0**.**72**				
	Asian	**−0**.**52**				
	African	**−0**.**88**				
Preference	Caucasian	**−0**.**52**	**0**.**96**			
	Asian	0.29	**0**.**99**			
	African	**−0**.**87**	**0**.**97**			
Brightness	Caucasian	**−0**.**69**	**0**.**95**	**0**.**92**		
	Asian	−0.22	**0**.**87**	**0**.**90**		
	African	**−0**.**80**	**0**.**66**	**0**.**59**		
Whiteness	Caucasian	**0**.**65**	**0**.**97**	**0**.**91**	**0**.**94**	
	Asian	−0.08	**0**.**87**	**0**.**83**	**0**.**76**	
	African	−0.41	0.18	0.38	**0**.**73**	
Transparency	Caucasian	**−0**.**68**	**0**.**99**	**0**.**95**	**0**.**89**	**0**.**93**
	Asian	−0.33	**0**.**95**	**0**.**92**	**0**.**69**	**0**.**96**
	African	**−0**.**83**	**0**.**92**	**0**.**97**	**0**.**86**	0.47

Caucasian *N *= 14						
Evaluation item	Stimulus type	Tone	Healthiness	Preference	Brightness	Whiteness
Healthiness	Caucasian	0.25				
	Asian	**−0**.**66**				
	African	**−0**.**67**				
Preference	Caucasian	**0**.**53**	**0**.**82**			
	Asian	0.52	**0**.**97**			
	African	**−0**.**63**	**0**.**97**			
Brightness	Caucasian	0.18	**0**.**82**	**0**.**46**		
	Asian	−0.20	**0**.**86**	**0**.**81**		
	African	**−0**.**66**	**0**.**94**	**0**.**99**		
Whiteness	Caucasian	**0**.**56**	**0**.**62**	**0**.**65**	**0**.**72**	
	Asian	0.15	0.40	0.32	0.45	
	African	0.47	−0.31	−0.07	−0.01	
Transparency	Caucasian	**0**.**53**	**0**.**79**	**0**.**54**	**0**.**88**	**0**.**78**
	Asian	0.48	−0.34	−0.27	0.00	0.29
	African	−0.26	−0.22	−0.39	−0.40	**−0**.**76**

a Results for each of the three skin-color types are shown separately.

Data with absolute values greater than or equal to 0.5 are marked in bold, and negative values are marked with a gray shade (continue on next page).

### General Experimental Results

Figure 4 shows the mean results with standard errors for each group's evaluation scores for the six items for the faces with the three skin-color types. For the skin tone evaluation item, observers were required to judge whether one of the presented faces was reddish (responding with a lower score) or yellowish (responding with a higher score). The skin tone results showed that the change in skin color (reddish-yellowish axis) perceived by the observers was consistent with the colorimetric hue change of the image stimulus: the reddish face was assigned a lower skin tone score, across all four observer groups. This indicated that the observers could correctly discriminate colorimetric hue changes in the face images.

This study aimed to observe the effects of perceived changes in skin color on other impressions. Figure 5 shows the results of the four groups without separating the face stimuli with different skin-color types. The slope of the linear approximation was then calculated. Table 3 shows the slope of the relationship between the pairwise average scores for all items. The first row shows the slopes of the data in Figure 5 (the relationship between the skin tone and other items). All groups, except the Thai observers, demonstrated a similar tendency, wherein faces rated with more redness were rated higher in other aspects. Regarding the impact of skin color changes on other impressions, most items showed a negative correlation (obtaining lower scores for skin tone and higher scores on other items.) or a weak positive correlation. Additionally, there was a positive correlation observed between all observers’ scores for each combination of healthiness, preference, brightness, whiteness, and transparency. This suggests that a subset of face perceptions was consistent across the ethnic groups investigated in this experiment. Compared with the other three groups, Thai observers assigned a narrower range of scores when evaluating skin tone. This may have contributed to the flattening of the average results.

Figure 6 shows the relationship between the skin tone (reddish-yellowish) and healthiness scores assigned by the four groups of observers. The results for each of the three skin-color types are presented separately. A negative correlation in the graph indicates that when observers evaluated an image stimulus as reddish, they also evaluated it as healthier. In other words, the reddishness and healthiness impressions of faces were positively correlated among Asian observers.

In the next subsection, we analyzed the slopes and correlation coefficients of the linear approximation lines associated with skin tone and other item scores for the four observer groups and three skin-color types.

### Skin-Color Variations Affect Impression Perception

To facilitate an overall comparison, Figure 7 shows the slope value for the relationship between skin tone and other items for the four observer groups. A positive slope indicates that as skin color was rated more yellowish, the other items were rated higher. Conversely, a negative slope indicated that other items were rated higher, as skin color was rated as more reddish.

The Japanese observers consistently highly rated reddish faces (obtaining lower scores for reddish skin on the item Tone and higher scores on other items), except for the whiteness of Caucasian and Asian faces. Other groups showed similar trends; however, the trends were less clear than those of the Japanese observers. This implies that Japanese observers may be more sensitive to changes in the face or skin, compared with observers from other ethnic groups.

Chinese observers showed a trend similar to that of Japanese observers in terms of the healthiness and preference evaluation of the Asian or African skin type. This may indicate that Japanese and Chinese observers associate reddish faces with other positive impressions, such as healthier and more preferred faces. This is similar to the results of the healthiness evaluated by Thai observers.

Thai, Chinese, and Caucasian observers did not show similar results for various skin-color types and items. Interestingly, Caucasian observers associated yellowish faces with positive impressions when evaluating own ethnicity faces, such as healthier and more preferred faces. Caucasians differed significantly in evaluating their preferences for Caucasian and Asian skin-color stimuli.

Table 4 shows the correlation coefficients among all evaluation items. The results for each of the three skin-color types are presented separately. Japanese observers showed higher correlations between skin tone and other items. Regardless of the observer group, strong correlations were found between healthiness and preference.

The slope values of the correlations between skin tone and other item evaluation scores for all observers were used for the ANOVA. Two-way ANOVA was conducted separately on the slope results of the four groups, and no significant differences were found in the skin-color type factor for all groups (Japanese [*F* [2, 359] = 0.435, *p *= .648, *η_p_*^2 ^= .013], Chinese [*F* [2, 179] = 0.202, *p *= .818, *η_p_*^2 ^= .029], Thai [*F* [2, 479] = 0.659, *p *= .518, *η_p_*^2 ^< .01], and Caucasian [*F* [2, 209] = 1.160, *p *= .316, *η_p_*^2 ^= .047]). Table 5 shows the *p*-values of the one-way ANOVA between the observer groups. Considering that different observer groups may have different criteria for evaluating different skin-color types (e.g., own ethnicity or other ethnicity), each of the three skin-color types was analyzed separately. There were no significant differences between the observer groups for the three skin-color types in terms of brightness, whiteness, and transparency, as in the preference evaluation for the African skin type. Shaffer's post hoc test was conducted for the significantly different results. Regarding the healthiness evaluation, there was a significant difference between the Japanese and Thai observers (*F* [1, 55] = 7.114, *p *= .010, *η_p_*^2 ^= .116) for Caucasian skin-color type. There were significant differences between Japanese and Thai observers (*F* [1, 55] = 11.148, *p *= .002, *η_p_*^2 ^= .171) and between Japanese and Caucasian observers (*F* [1, 37] = 7.184, *p *= .011, *η_p_*^2 ^= .166) for Asian skin-color type. There was a significant difference between Japanese and Thai observers (*F* [2, 55] = 8.638, *p *= .005, *η_p_*^2 ^= .138) and between Japanese and Caucasian observers (*F* [2, 37] = 7.313, *p *= .010, *η_p_*^2 ^= .169) for the African skin-color type.

**Table 5.** Results of the *p*-Values of the One-Way Analysis of Variance Between the Four Observer Groups (*p < 0.05).

Skin-color type	Evaluation item				
	Healthiness	Preference	Brightness	Whiteness	Transparency
Caucasian skin-color	.012 *	.027 *	.415	.716	.128
Asian skin-color	.006 *	.003 *	.502	.988	.060
African skin-color	.013 *	.436	.495	.149	.175

Regarding the preference evaluation, there was a significant difference between Japanese and Caucasian observers (*F* (1, 37) = 6.379, *p *= .016, *η_p_*^2 ^= .127) for Caucasian skin-color type and between Japanese and Thai observers (*F* (1, 55) = 16.070, *p *< .001, *η_p_*^2 ^= .229) for Asian skin-color type.

## Discussion

We investigated the effect on facial impressions when skin color varied on the red-yellow axis for six face types (three male and three female faces). The observers were East Asians and Caucasians. The results from the Japanese observers showed that reddish skin was positively correlated with other evaluation items under almost all conditions. For Chinese observers, under stimulus conditions consistent with the skin color of their own ethnic group and African skin color, reddish skin was positively correlated with the healthiness and preference evaluation items. In contrast, for Caucasian observers, under stimulus conditions consistent with the skin color of their own ethnic group, the impression of reddish skin was negatively correlated with healthiness and preference. These results suggest that the effects of skin-color variations on the perceived impressions may be inconsistent across ethnic groups. The reason for this may be that skin color is inherently diverse.

Chinese observers rated Asian and African stimuli as redder, healthier, and more preferred. This result was similar to that reported by Tan and Stephen (2019). Japanese and Thai observers showed similar results. In contrast, when Caucasians evaluated their own ethnicity stimuli (Caucasian-type stimuli), they evaluated yellowish faces as healthier and more preferred. There were inconsistencies in their evaluations of faces of other races. Studies on Caucasian observers have shown that increased facial skin yellowness (CIELAB b*) and lightness (L*) appear healthy in Caucasian faces to this population. Carotenoid color has been identified as a valid indicator of human health (Stephen et al., 2011).

The skin-color variations we used in this experiment are somewhat limited in that we only used ±8 based on the average hue for each skin-color type. We cannot rule out the possibility that the range of skin-color changes was insufficient to cause perceptual changes in a specific ethnic group. For example, some observers reported that the skin-color change range used in this experiment was not obvious for the African stimulus type. This may explain the relatively large difference among observer groups. Different groups of observers may differ in their sensitivity to the perception of faces from different ethnicities.

The three types of faces—Caucasian, Asian, and African—used in this study were created by averaging faces from a face database; however, the numbers of faces used for averaging differed in each group because the content of the database limited the number of original face images used for synthesis. Considering that faces averaged from a larger number of face images possess higher averageness, it may not be possible to exclude that the uniformity and homogeneity of the face can be factors affecting the results, such as preference.

Although the monitor used in the experiments conducted in UK exhibited noticeably higher luminance, we considered the influence to be small because we did not find a significant difference between observers in the UK (*N *= 11) and France (*N *= 3). Moreover, we investigated the correlations between changes in skin color and other parameters. Therefore, the luminance difference did not affect the results.

**Figure 8.**
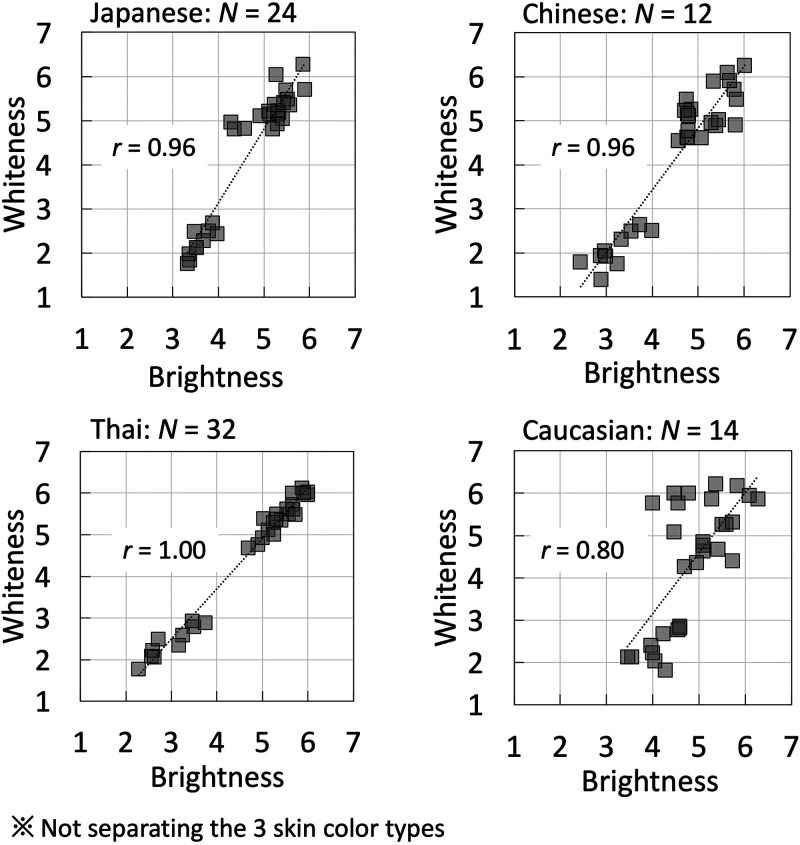
The relationship between brightness and whiteness for the four observer groups. The horizontal axis shows the evaluated brightness score, and the vertical axis shows the whiteness score.

He et al. (2021) showed that Japanese observers matched reddish faces with higher lightness, Thai observers matched yellowish faces with higher lightness, and Chinese observers presented different results for different skin-color types. Differences in experimental methods (e.g., absolute judgments and relative judgments) were reported by He et al. (2024) to affect observers’ brightness judgments of faces. In the absolute judgment, similar tendencies were observed between the observer groups. The results of the impression ratings of the absolute judgments used in this study were similar to those of Japanese judgments of whiteness and brightness (Yoshikawa et al., 2012, He et al., 2021). Figure 8 shows the correlation between brightness and whiteness for the different observer groups. All four observer groups showed strong correlations. This indicates that the judgment criteria for whiteness and brightness have cross-cultural similarities.

In addition, when instructions for the experimental task were given, the observers were asked to evaluate the perceived brightness and whiteness levels, and no observer questioned the differences between the two parameters. As shown in Figure 8, there was a high correlation between the evaluation results of the two items, and it can perhaps be hypothesized that the criteria of brightness and whiteness are intended to be close when perceiving skin color. This is consistent with Yoshikawa et al. (2012)'s view that whiteness is more suitable for the expression of facial skin-color change from light to dark because it is commonly used in facial skin evaluation. However, the possibility that the observers did not achieve complete differentiation cannot be ruled out. A more exhaustive investigation is required in the future. However, impressions of faces still appeared to be influenced by impressions of ethnic backgrounds between East Asian and Caucasian groups.

Interestingly, the independent results for the three skin-color types were partially inconsistent with the average results. This may provide evidence that different judgment criteria are used when perceiving the impressions of faces from one's own ethnic group versus those from other ethnic groups. Our results highlight the necessity for independent analyses of face shape and skin-color type for stimulus images in the study of facial impressions.

### Conclusion

In our cross-cultural exploration, we investigated the impact of skin-color variations along with the red-yellow axis on the facial impressions of six representative face shapes. Our approach focused on isolating the hue angle of facial skin color as the sole variable, thus allowing us to discern the unique influence of skin color itself. Notably, East Asian cultures link reddish skin tones with positive attributes, whereas Caucasians associate positive traits with yellowish skin. Our results demonstrate that specific cultural backgrounds influence the perception of human faces. In advertising, healthcare, or interpersonal relationships, understanding and respecting diverse cultural viewpoints can foster positive interactions and mutual understanding. By delving deeper into how culture shapes skin-color perception, we can encourage more inclusive and culturally attuned practices in an increasingly globalized world.
